# Interaction between CD177 and platelet endothelial cell adhesion molecule-1 downregulates membrane-bound proteinase-3 (PR3) expression on neutrophils and attenuates neutrophil activation induced by PR3-ANCA

**DOI:** 10.1186/s13075-018-1710-0

**Published:** 2018-09-20

**Authors:** Hui Deng, Nan Hu, Chen Wang, Min Chen, Ming-Hui Zhao

**Affiliations:** 1 Renal Division, Department of Medicine, Peking University First Hospital, Peking University Institute of Nephrology, Beijing, 100034 China; 2 Key Laboratory of Renal Disease, Ministry of Health of China, Beijing, 100034 China; 3 Key Laboratory of Chronic Kidney Disease Prevention and Treatment, Ministry of Education, Peking University, Beijing, 100034 China; 4 Peking-Tsinghua Center for Life Sciences, Beijing, 100034 China

**Keywords:** ANCA, Proteinase-3, CD177, Platelet endothelial cell adhesion molecule-1

## Abstract

**Background:**

A recent study found that CD177 served as a receptor of membrane-bound proteinase-3 (mPR3) in a subset of neutrophils. Furthermore, CD177 has been identified as a high-affinity heterophilic binding partner for the endothelial cell platelet endothelial cell adhesion molecule-1 (PECAM-1). The current study aimed to investigate whether the interaction between PECAM-1 and CD177 could influence mPR3 expression as well as PR3-antineutrophil cytoplasmic antibody (ANCA)-induced neutrophil activation and glomerular endothelial cell (GEnC) injury.

**Methods:**

The effect of interaction between CD177 and PECAM-1 on mPR3 expression was explored by enzyme-linked immunosorbent assay (ELISA) and flow cytometry. The effect of PECAM-1 on neutrophil activation and GEnC injury induced by PR3-ANCA-positive immunoglobulin (Ig)Gs was evaluated by dihydrorhodamine (DHR) assay and ELISA. CD177-negative neutrophils were selected by magnetic cell sorting (MACS), and the inhibitory effect of PECAM-1 on CD177-negative and mixed neutrophils was explored by measuring neutrophil degranulation.

**Results:**

The level of specific interaction between CD177 and PECAM-1 was elevated with increasing CD177 concentration. The expression of mPR3 significantly decreased in neutrophils preincubated with PECAM-1 in a dose-dependent manner. Consistently, the levels of respiratory burst and degranulation induced by PR3-ANCA-positive IgGs in recombinant human tumor necrosis factor-alpha (TNF-α)-primed neutrophils was significantly reduced by preincubation with PECAM-1 (440.6 ± 123.0 vs. 511.4 ± 95.5, *p* < 0.05; and 3155.0 ± 1733.0 ng/ml vs. 5903.0 ± 717.5 ng/ml, *p* < 0.05, respectively). In CD177-negative neutrophils incubated with PR3-ANCA-positive IgGs, the level of degranulation was not significantly changed by preincubation with PECAM-1. However, in mixed neutrophils, PECAM-1 significantly decreased the level of degranulation induced by PR3-ANCA-positive IgGs (1015.9 ± 229.2% vs. 1725.2 ± 412.4%, *p* < 0.01). Furthermore, with preincubation of TNF-α-primed neutrophils with PECAM-1, the level of soluble intercellular cell adhesion molecule-1 (sICAM-1), a marker of endothelial cell activation and injury, in the supernatant of GEnCs treated with primed neutrophils plus PR3-ANCA-positive IgGs was significantly attenuated (112.7 ± 24.2 pg/ml vs. 167.5 ± 27.7 pg/ml, *p* < 0.05).

**Conclusions:**

PECAM-1 can decrease the level of mPR3 expression on neutrophils, resulting in attenuation of neutrophil activation and subsequent GEnC injury induced by PR3-ANCA-positive IgGs.

## Background

Antineutrophil cytoplasmic antibody (ANCA)-associated vasculitis (AAV) consists of granulomatosis with polyangiitis (GPA, previously named Wegener’s granulomatosis), microscopic polyangiitis (MPA), and eosinophilic granulomatosis with polyangiitis (EGPA) [1]. The kidney is one of the most commonly involved organs in AAV. ANCAs, the serological markers for primary small vessel vasculitis, are involved in inducing and amplifying endothelial injury in AAV [2]. Proteinase-3 (PR3) and myeloperoxidase (MPO) are the two main target antigens of ANCA in AAV [3, 4]. During the priming process of neutrophils by proinflammatory cytokines such as tumor necrosis factor-alpha (TNF-α), membrane-bound PR3 (mPR3) is upregulated in a subset of neutrophils [5]. PR3-ANCA may recognize mPR3 and lead to degranulation and reactive oxygen species (ROS) production in neutrophils, causing massive injury to endothelial cells, in particular glomerular endothelial cells (GEnCs) [5, 6].

CD177 is a neutrophil surface molecule that was identified in 1971 as the target of alloimmune antibodies associated with fetal neutropenia [7]. Its expression is restricted to a subset of neutrophils and the percentage of CD177-positive neutrophils ranges from 0% to 100% in an individual, with a mean percentage of 45–65% [8–10]. The function of CD177 is largely unknown. Recently, CD177 has been identified as a high-affinity heterophilic binding partner for platelet endothelial cell adhesion molecule-1 (PECAM-1)/CD31 on endothelial cells [11]. PECAM-1 is highly expressed on endothelial cells and is a major constituent of the endothelial cell intercellular junction in confluent vascular beds [12, 13]. Interaction of CD177 and PECAM-1 has indicated its role as an adhesion molecule in neutrophil adhesion and transmigration.

As previously mentioned, in the pathogenesis of endothelium injury in AAV, PR3-ANCA recognizes mPR3 and triggers degranulation and respiratory burst of neutrophils, which in turn causes necrosis of endothelial cells [14]. CD177 and mPR3 are colocalized on the neutrophil membrane, and CD177 is probably the receptor for mPR3 [15–17]. Therefore, it is of interest to investigate the role of CD177 as the receptor of mPR3, and its binding partner PECAM-1, in the process of neutrophil activation. We hypothesized that the interaction of CD177 and PECAM-1 may influence the binding of mPR3 to CD177 on the neutrophil membrane. Furthermore, PR3-ANCA-mediated neutrophil activation and endothelial injury may also be affected by the interaction between CD177 and PECAM-1 on neutrophils.

## Methods

### Reagents

Recombinant PECAM-1, junctional adhesion molecule-1 (JAM-1), and CD177 were purchased from Sino Biological Inc. (Beijing, China). Fluorochrome dihydrorhodamine (DHR), phorbol myristate acetate (PMA), and normal human immunoglobulin (Ig)G were purchased from Sigma (St Louis, USA). For indirect enzyme-linked immunosorbent assay (ELISA), mouse anti-human CD177 antibody was purchased from Sino Biological Inc. and horseradish peroxidase (HRP)-conjugated goat anti-mouse IgG was purchased from Abcam (Cambridge, UK). For flow cytometry analysis, phycoerythrin (PE)-conjugated mouse monoclonal antibody against human CD177 and the isotype control mouse IgG1 were purchased from BioLegend (San Diego, CA, USA). Fluorescein isothiocyanate (FITC)-conjugated mouse monoclonal antibody against human PR3 and the isotype control mouse IgG1 were purchased from Abcam. For magnetic neutrophil sorting, anti-PE microbeads and separation columns were purchased from Miltenyi Biotech (Bergisch-Gladbach, Germany). For Western blot, antibodies against SHP-1 (C14H6) and phosphor-SHP-1 (Tyr564) were purchased from Cell Signaling Technology (Boston, MA, USA), and mouse anti-human GAPDH antibody was purchased from Santa Cruz Biotech (Santa Cruz, CA, USA).

### Cell culture

Primary human renal GEnCs (ScienCell Research Laboratories, San Diego, CA, USA) were cultured in endothelial cell basal medium (ECM) (ScienCell) with the addition of 5% fetal bovine serum (FBS), 1% penicillin/streptomycin, and 1% endothelial cell growth factor for the formation of a confluent endothelial cell monolayer. The flasks for cell subculture were bio-coated with human plasma fibronectin (Millipore, Billerica, USA) beforehand according to the manufacturer’s recommendations. For synchronization of the cell cycle, GEnC monolayers were starved in basal medium without serum and endothelial cell growth factor for 12 h without bio-coating. All experiments were performed using GEnCs at passage 3–5. All cultures were incubated at 37 °C in 5% CO_2_.

### Interaction between CD177 and PECAM-1

The interaction between CD177 and PECAM-1 was detected by ELISA with recombinant soluble PECAM-1 (sPECAM-1) at 2 μg/ml as the solid-phase antigen. sPECAM-1 in a coating buffer (0.05 M bicarbonate buffer, pH 9.6) was used to coat the wells of half of a polystyrene microtiter plate (Nunc-Immuno plate; Nunc, Roskilde, Denmark) and was incubated overnight at 4 °C. The other half of the plate was coated with coating buffer alone to establish antigen-free wells. The wells were blocked with 3% bovine serum albumin (BSA) in phosphate-buffered saline (PBS) and incubated with CD177 at various concentrations for 1 h at 37 °C. After washing three times with PBS-tween 20 (PBS-T), the wells were incubated with mouse anti-human CD177 antibody (1:1000; Sino Biological) for 1 h at 37 °C, and HRP-conjugated goat anti-mouse IgG (1:1000) was used as the secondary antibody. Tetramethylbenzidine (TMB; Sigma, St. Louis, MO) was used as the substrate and the reaction was stopped by the addition of sulfuric acid 0.5 mol/L (Carl Roth GmbH, Germany). Optical densities of formed complexes were measured at 450 nm using a microplate reader (Bio-Rad, Tokyo, Japan).

### Isolation and priming of neutrophils

Neutrophils were isolated as described previously [18]. In brief, venous human blood for neutrophil isolation was obtained from healthy donors by venipuncture and anticoagulated with ethylenediaminetetraacetic acid (EDTA). Neutrophils were isolated by density gradient centrifugation on Lymphoprep (Nycomed, Oslo, Norway). Erythrocytes were lysed with ice-cold red cell lysis buffer (Tiangen Biotech, Beijing, China), and then neutrophils were washed in PBS without Ca^2+^/Mg^2+^ (PBS^−/−^; Chemical reagents, Beijing, China) and suspended in PBS^−/−^ to a concentration of 1 × 10^6^ cells/ml and used for further analysis. The trypan blue staining technique was used as an index of the proportion of viable cells in a cell population. Where indicated, cells were primed with 2 ng/ml recombinant TNF-α (Sigma, USA) at 37 °C for 15 min, and untreated cells were incubated with control medium under the same conditions.

### Magnetic neutrophil sorting

CD177-negative neutrophils were separated with negative selection by magnetic cell sorting (MACS) separation columns (Miltenyi Biotech, Bergisch-Gladbach, Germany) according to the manufacture’s manual, as described previously [19]. All steps were carried out on ice. Freshly isolated neutrophils were stained with PE-conjugated monoclonal antibody to CD177 (MEM166). Subsequently, the cells were labeled with anti-PE microbeads (Miltenyi Biotech) and loaded on MACS LD columns (Miltenyi Biotech). The flow-through containing the nonlabeled CD177-negative neutrophils was collected. The purity of CD177-negative neutrophils was 86.4 ± 8.5% as assessed by flow cytometry.

### Preparation of PR3-ANCA-positive IgGs

PR3-ANCA-positive IgGs were prepared from plasma exchange liquid of patients with active PR3-ANCA-positive primary small vessel vasculitis using a High-Trap-protein G column on an AKTA-FPLC system (GE Biosciences, South San Francisco, CA, USA). The preparation of IgGs was performed according to methods described previously [20]. In brief, plasma exchange liquid was filtered through a 0.2-μm syringe filter (Schleicher & Schuell, Duesseldorf, Germany) and applied to a High-Trap-protein G column on an AKTA-FPLC system (GE Biosciences). The column was treated with equal volume of 20 mmol/l Tris-HCl buffer, pH 7.2 (binding buffer), and IgG was eluted with 0.1 mol/l glycine-HCl buffer, pH 2.7 (elution buffer). After the antibodies emerged from the column, the pH value of the eluent was adjusted to pH 7.0 using 2 mol/l Tris-HCl (pH 9.0) immediately. The protein concentration of the antibodies was measured using the Nanodrop-1000 (Pierce, Rockford, IL, USA), and the level of PR3-ANCA IgG was measured by an ELISA kit (EUROIMMUN, Lubeck, Germany). We obtained written informed consent from the participants involved in our study. The research was in compliance with the Declaration of Helsinki and was approved by the clinical research ethics committee of the Peking University First Hospital.

### Detection of mPR3 expression on neutrophils and PR3 in supernatant after incubation with PECAM-1

**Fig. 1:**
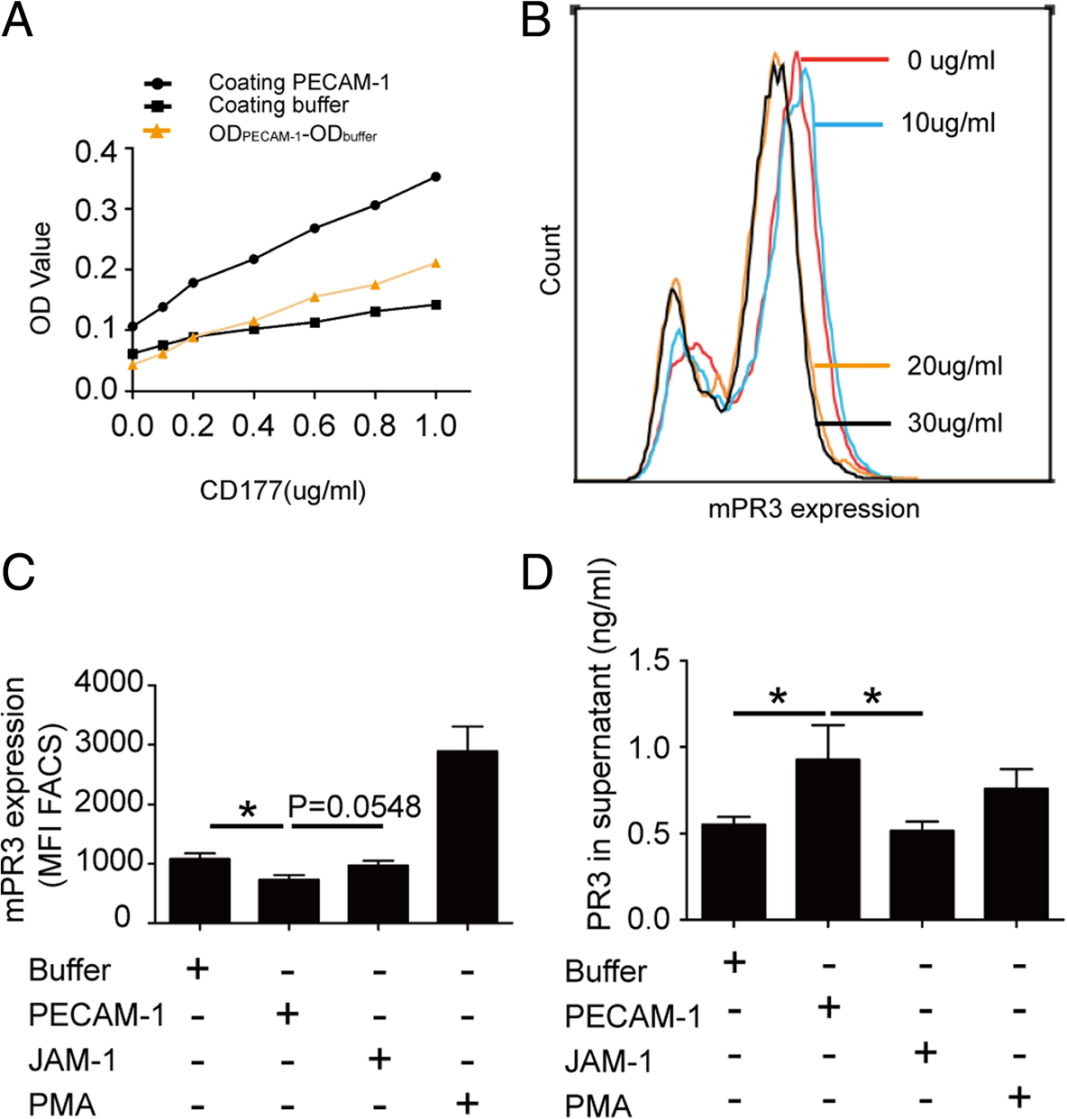
Downregulation of mPR3 after treating neutrophils with PECAM-1. **a** interaction between platelet endothelial cell adhesion molecule-1 (PECAM-1) and CD177 at various concentrations. **b** Representative histogram of the effect of PECAM-1 on membrane-bound proteinase-3 (mPR3) expression in a dose-dependent manner. **c** Incubation of TNF-α-primed neutrophils with PECAM-1 at 30 μg/ml significantly decreased mPR3 expression. Bars denote means ± SD of mPR3 expression (mean fluorescence intensity; MFI). **d** Incubation of TNF-α-primed neutrophils with PECAM-1 at 30 μg/ml significantly increased proteinase-3 (PR3) levels in the supernatant. Bars denote means ± SD of PR3 concentration (ng/ml). Neutrophils treated with phorbol myristate acetate (PMA) were employed as positive control. *p < 0.05. FACS fluorescence-activated cell sorting, JAM-1 junctional adhesion molecule-1, OD optical density

Expression of mPR3 on neutrophils was detected by flow cytometry. Primed neutrophils were incubated with PECAM-1 at serial concentrations (Fig. 1b). There was a dose-dependent response of PECAM-1 in inducing mPR3 downregulation. In further experiments, neutrophils were incubated with PECAM-1 at a concentration of 30 μg/ml or buffer control for 2 h at 37 °C. Since PECAM-1 and JAM-1 are both members of the immunoglobulin superfamily of adhesion molecules with many similarities in their expression profiles and functions [21, 22], JAM-1 was used as the control. Levels of PR3 in the supernatant were tested using commercially available ELISA kits (Elabscience, Wuhan, China). The assay was conducted according to the manufacturer’s instructions. All further steps of mPR3 detection on neutrophils were performed on ice, and washing steps were performed using PBS. TruStain FcR Solution (BioLegend, San Diego, CA, USA) was used in all samples prior to the addition of antibodies to block nonspecific binding. Next, cells were stained with a saturating dose of FITC-conjugated mouse monoclonal antibody directed against human PR3 (Abcam, Cambridge, UK) or with isotype antibody for 20 min in the dark. Fluorescence intensity of FITC was analyzed using flow cytometry. Samples were analyzed using a FACScan (BD, Biosciences, USA). Neutrophils were gated in forward/sideward scatter (FSC/SSC) and data were collected from 10,000 cells per sample. Data were analyzed using FlowJo software (TreeStar, Ashland, Oregon, USA).

### Evaluation of neutrophil respiratory burst by DHR assay

We assessed the generation of ROS using DHR as described previously [23]. This method is based on the fact that reactive oxygen radicals cause an oxidation of the nonfluorescent DHR to the green fluorescent rhodamine. In brief, isolated neutrophils suspended in Hanks’ balanced salt solution (HBSS) were incubated with 0.05 mM DHR123 (Sigma-Aldrich, Louis, USA) for 30 min at 37 °C. Sodium azide (NaN_3_; 2 mM) was added to prevent intracellular breakdown of H_2_O_2_ by catalase. The neutrophils were then primed with TNF-α (2 ng/ml) for 15 min at 37 °C. After incubating with PECAM-1 (30 μg/ml) or JAM-1 (30 μg/ml) as described above, patient-derived ANCA-positive IgGs (5 RU/ml) or normal IgG were added. After incubation at 37 °C for 1 h, the reaction was stopped by the addition of 1 ml ice-cold 1% BSA in HBSS. Samples were kept on ice and analyzed using a FACScan. Neutrophils were gated in FSC/SSC and mean fluorescence intensity (MFI; representing the level of neutrophil activation) were collected from 10,000 cells per sample. Data were analyzed using FlowJo software (TreeStar, Ashland, Oregon, USA).

### Measurement of neutrophil degranulation by lactoferrin quantification

Lactoferrin, an iron-binding multifunctional glycoprotein, is an abundant component of the specific granules of neutrophils [24, 25]. Lactoferrin is considered as a biomarker of neutrophil degranulation [26–28]. Neutrophils were primed with TNF-α (2 ng/ml) at 37 °C for 15 min, and then were incubated with PECAM-1 or JAM-1 (30 μg/ml) at 37 °C for 2 h followed by stimulation with patient-derived ANCA-positive IgGs or normal IgG for 1 h. Lactoferrin in the supernatant was tested by ELISA using a commercial kit (Abcam, Cambridge, UK). The ELISA procedure for measuring lactoferrin was performed according to the manufacturer’s instructions, as described previously [27].

### SHP-1 phosphorylation detected by Western blot

To detect phosphor-SHP-1, isolated neutrophils were primed with TNF-α (2 ng/ml) or PBS at 37 °C for 15 min, and then were incubated with or without PECAM-1 (30 μg/ml) for 1 h. The cells were then incubated on ice in cell lysis buffer (Beyotime Biotechnology, Beijing, China) supplemented with proteinase inhibitors (Sigma, St. Louis, MO, USA) and phosphatase inhibitors (Roche, Mannheim, Germany) for 30 min. The insoluble material was pelleted, and samples were boiled with reduced loading buffer and run in 8% sodium dodecyl sulfate-polyacrylamide gel electrophoresis (SDS-PAGE) gels. Protein was transferred to polyvinylidene difluoride (PVDF) membranes (Millipore, Bedford, MA, USA), and detected by rabbit anti-human SHP-1 antibody (1:1000), rabbit anti-human phosphor-SHP-1 antibody (1:1000), and mouse anti-human GAPDH antibody (1:500) overnight at 4 °C. Finally, the strips were incubated with HRP-conjugated goat anti-mouse (1:5000) or goat anti-rabbit secondary antibodies (1:5000) for 1 h at room temperature with gentle agitation and then revealed on autoradiographic film using the ECL Plus Western Blotting Detection System (GE Healthcare, Piscataway, NJ, USA).

### GEnC activation and injury indicated by soluble intercellular cell adhesion molecule-1

Soluble intercellular cell adhesion molecule-1 (sICAM-1) is considered as one of the markers of endothelial cell activation and injury [29]. To explore the role of PECAM-1 in PR3-ANCA-induced endothelial cell injury, the isolated neutrophils were incubated with PECAM-1, JAM-1, or control buffer at 37 °C for 1 h after priming with TNF-α. After incubation, neutrophils were added to GEnC monolayers with patient-derived PR3-ANCA-positive IgGs or normal IgG. After incubation for 4 h at 37 °C, the cell culture supernatant was collected for the ICAM-1 assays. Samples were tested using the human ICAM-1/CD54 ELISA kit (R&D, Abingdon, UK). The assay was conducted according to the manufacturer’s instructions as described previously [20]. In brief, samples were added to the microtiter plate coated with capture antibody and incubated for 2 h at room temperature, followed by detection antibody incubation for another 2 h. Then HRP-conjugated streptavidin was added. After 20 min of incubation avoiding direct light, the plate was washed, and substrate solution was added to the wells. After adding the stop solution, the absorption measurements were obtained at 450 nm (with a correction of 570 nm to eliminate optical imperfections in the plate) using a microtiter plate reader (Bio-Rad iMark™ Microplate Reader). All samples and standards were performed in duplicate.

### Statistical analysis

The Shapiro-Wilk test was used to examine whether the data were normally distributed. Quantitative data are expressed as mean ± SD for data that was normally distributed or the median and range for data that was not normally distributed. Differences in quantitative parameters between groups were assessed using one-way analysis of variance (ANOVA) for data that was normally distributed or the Mann-Whitney *U* test for data that was not normally distributed, as appropriate. Differences were considered significant if *p* < 0.05. Analysis was performed with SPSS statistical software package (version 13.0, Chicago, IL, USA).

## Results

### Interaction between CD177 and PECAM-1

To explore the interaction between CD177 and PECAM-1, indirect ELISA was performed using soluble PECAM-1 (sPECAM-1) and CD177 at various concentrations. As shown in Fig. 1a, the level of specific interaction between CD177 and PECAM-1, indicated by OD_PECAM1_ – OD_buffer_, elevated with increasing CD177 concentration in a dose-dependent manner.

### Downregulation of mPR3 induced by the interaction between PECAM-1 and CD177 on neutrophils

Neutrophils were preincubated with serial concentrations of sPECAM-1 (0, 10, 20, and 30 μg/ml) after priming. Expression of mPR3 on neutrophils was analyzed using flow cytometry. The level of mPR3 gradually decreased with increased concentration of sPECAM-1 (Fig. 1b). After priming with TNF-α, mPR3 expression significantly decreased by treating with sPECAM-1 at 30 μg/ml (730.1 ± 228.8 vs. 1082.0 ± 267.4, *p* < 0.05). Treating neutrophils with JAM-1, another adhesion molecule on endothelial cells, at 30 μg/ml did not significantly affect mPR3 expression (970.4 ± 229.8 vs. 1082.0 ± 267.4, *p* = 0.38). Neutrophils activated by PMA showed high levels of mPR3, which was used as the positive control (Fig. 1c).

PR3 in the supernatant was detected by ELISA. In primed neutrophils treated with sPECAM-1, the concentration of PR3 in supernatant was significantly higher than that treated with JAM-1 (0.93 ± 0.60 ng/ml vs. 0.52 ± 0.21 ng/ml, *p* < 0.05). However, the PR3 concentration was comparable between neutrophils treated with buffer and JAM-1 (0.55 ± 0.17 ng/ml vs. 0.52 ± 0.21 ng/ml, *p* = 0.6143) (Fig. 1d).

### PECAM-1 attenuated the ANCA-induced respiratory burst of neutrophils

**Fig. 2:**
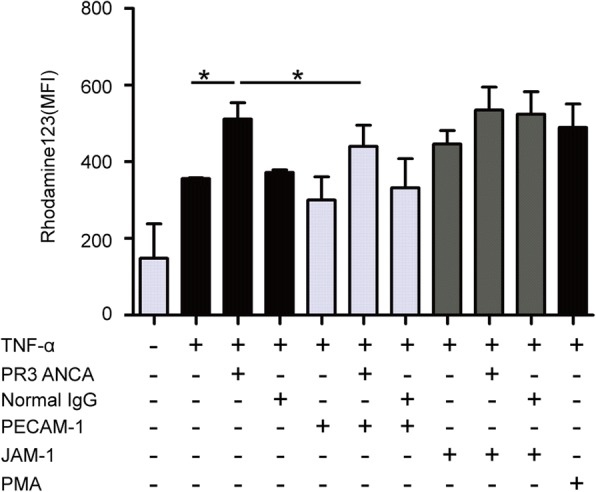
PECAM-1 incubation decreased antineutrophil cytoplasmic antibody (ANCA)-induced respiratory burst of neutrophils. Neutrophil respiratory burst detected by DHR assay was performed after proteinase-3 (PR3)-ANCA immunoglobulin (Ig)G incubation for 1 h. Neutrophils treated with phorbol myristate acetate (PMA) were employed as positive control. Bars denote means ± SD of Rhodamine 123 expression (mean fluorescence intensity; MFI). *p < 0.05. JAM-1 junctional adhesion molecule-1, PECAM-1 platelet endothelial cell adhesion molecule-1, PR3 ANCA PR3-ANCA-positive IgGs, TNF-α tumor necrosis factor-alpha

Compared with TNF-α-primed neutrophils, the MFI value of rhodamine was significantly higher in TNF-α-primed neutrophils treated with PR3-ANCA-positive IgGs (511.4 ± 95.5 vs. 356.7 ± 2.3, *p* < 0.05) (Fig. 2), and the MFI value in TNF-α-primed neutrophils was comparable with neutrophils treated with normal IgG (372.0 ± 11.8 vs. 356.7 ± 2.3, *p* = 0.0916) (Fig. 2). In the presence of PR3-ANCA-positive IgGs, the level of oxygen radical production significantly decreased in neutrophils preincubated with PECAM-1 (440.6 ± 123.0 vs. 511.4 ± 95.5, *p* < 0.05), while it did not significantly change by preincubation with JAM-1 (535.2 ± 134.1 vs. 511.4 ± 95.5, *p* = 0.7547) (Fig. 2).

### PECAM-1 decreased ANCA-induced degranulation of neutrophils

**Fig. 3:**
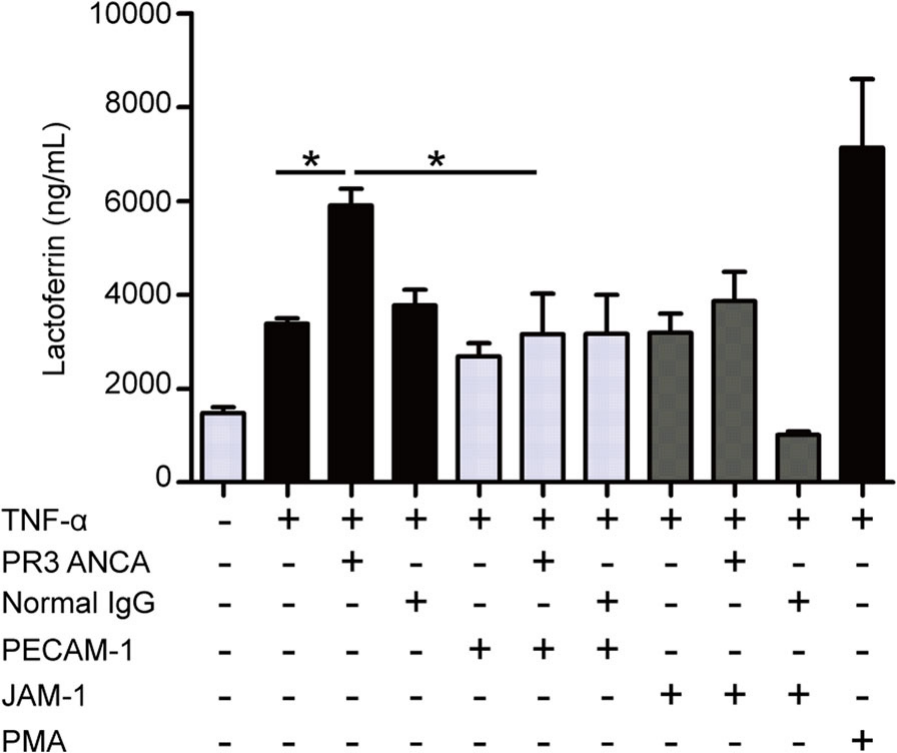
PECAM-1 incubation decreased antineutrophil cytoplasmic antibody (ANCA)-induced degranulation of neutrophils. Lactoferrin is considered as a biomarker of neutrophil degranulation and was detected after proteinase-3 (PR3)-ANCA immunoglobulin (Ig)G incubation for 1 h. Neutrophils treated with phorbol myristate acetate (PMA) were employed as positive control. Bars denote means ± SD of lactoferrin concentration (ng/ml). *p < 0.05. JAM-1 junctional adhesion molecule-1, PECAM-1 platelet endothelial cell adhesion molecule-1, PR3 ANCA PR3-ANCA-positive IgGs, TNF-α tumor necrosis factor-alpha

ANCA-induced neutrophil degranulation was determined by measuring the concentration of lactoferrin in the supernatant. Compared with TNF-α-primed neutrophils, the concentration of lactoferrin in the supernatant significantly increased in TNF-α-primed neutrophils treated with PR3-ANCA-positive IgGs (5903.0 ± 717.5 ng/ml vs. 3382 ± 233.0 ng/ml, *p* < 0.05), while the elevation of lactoferrin concentration was significantly inhibited by preincubation with PECAM-1 (3155.0 ± 1733.0 ng/ml vs. 5903.0 ± 717.5 ng/ml, *p* < 0.05) (Fig. 3).

### Effect of PECAM-1 on ANCA-induced degranulation in CD177-negative neutrophils

**Fig. 4:**
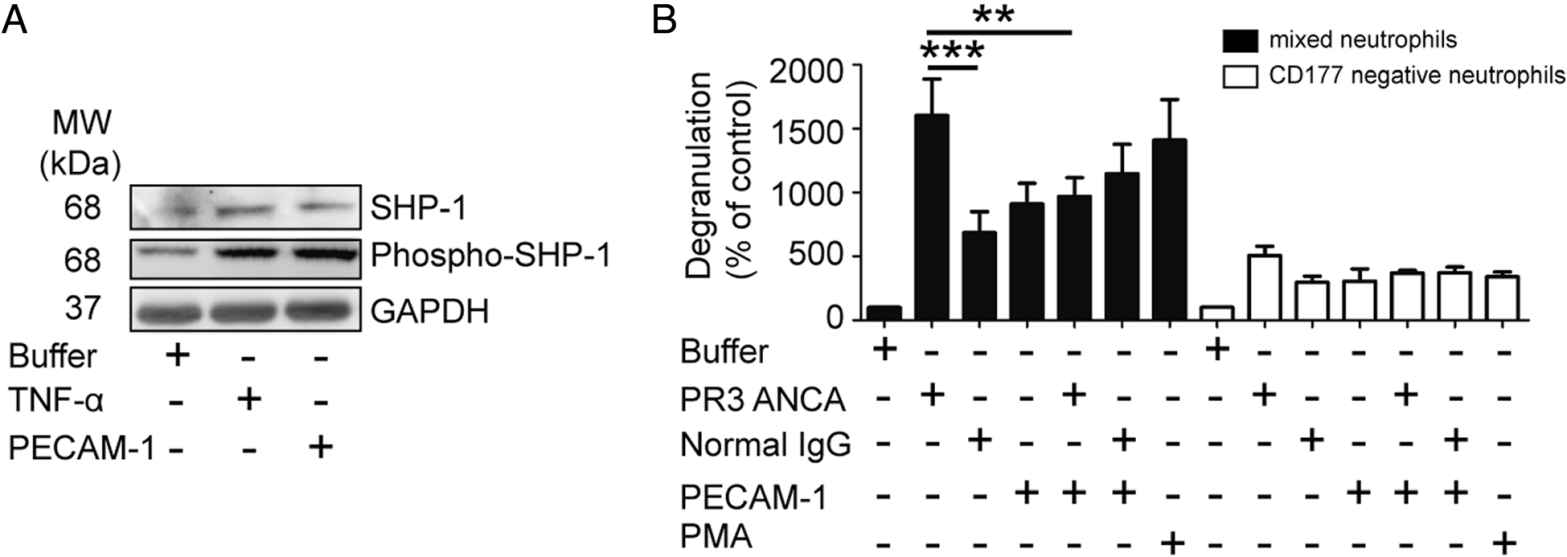
Effect of PECAM-1 on degranulation in CD177-negative neutrophils. **a** SHP-1 phosphorylation was detected in neutrophils incubated with platelet endothelial cell adhesion molecule-1 (PECAM-1). **b** Effect of PECAM-1 on degranulation of CD177-negative and mixed neutrophils. Degranulation induced by proteinase-3 (PR3)-antineutrophil cytoplasmic antibody (ANCA)-positive immunoglobulin (Ig)Gs (PR3 ANCA) was little influenced by preincubation with soluble PECAM-1 in CD177-negative neutrophils, but it was significantly inhibited by soluble PECAM-1 in mixed neutrophils. The level of lactoferrin was expressed as a percentage of control in each subset. The purity of the CD177-positive subset in mixed neutrophils was 72.7 ± 10.7% and the purity of CD177-negative neutrophils after selecting was 86.4 ± 8.5%. Bars represent mean ± SD of repeated measurements from four independent experiments. **p < 0.01, ***p < 0.001. MW molecular weight, PMA phorbol myristate acetate, TNF-α tumor necrosis factor-alpha

It has been reported that PECAM-1 has two immunoreceptor tyrosine-based inhibitory motifs (ITIMs). The homophilic interaction between PECAM-1 on neutrophils and endothelial cells could induce ITIM phosphorylation, resulting in inhibitory signal pathway activation, including the protein-tyrosine phosphatase SHP-1 phosphorylation [30, 31]. In our study, phosphor-SHP-1 was detected in neutrophils incubated with PECAM-1 (Fig. 4a), indicating the existence of a homophilic interaction of PECAM-1. We speculated that the detected inhibitory effect of PECAM-1 on neutrophil activation not only resulted from the heterophilic interaction between CD177 and sPECAM-1, but also from the homophilic interaction between transmembrane PECAM-1 and sPECAM-1. CD177-negative neutrophils were acquired by negative selection to test the effect of homophilic interaction of PECAM-1. The initial isolated mixed neutrophils without selection were assessed in parallel for comparison, and the samples contained on average 72.7 ± 10.7% CD177-positive neutrophils. In CD177-negative neutrophils incubated with PR3-ANCA, the level of degranulation did not significantly change by preincubation with PECAM-1, suggesting that the homophilic interaction of PECAM-1 has little, if any, inhibitory effect on neutrophil activation induced by PR3-ANCA. However, in mixed neutrophils, the level of degranulation induced by PR3-ANCA-positive IgGs was significantly higher than that induced by normal IgG (expressed as percentages of control in each subset, 1725.2 ± 412.4% vs. 878.4 ± 309.3%, *p* < 0.001). Preincubation with PECAM-1 significantly decreased the level of degranulation induced by PR3-ANCA-positive IgGs (1015.9 ± 229.2% vs. 1725.2 ± 412.4%, *p* < 0.01) (Fig. 4b). The inhibition rate of PECAM-1 to degranulation induced by PR3-ANCA was significantly higher in mixed neutrophils than that in CD177-negative neutrophils (38.5 ± 6.3% vs. 15.0 ± 13.0%, *p* < 0.05). These results indicated that the heterophilic interaction between CD177 and PECAM-1 contributed to the dominant inhibitory effect on neutrophil activation induced by PR3-ANCA.

### PECAM-1 decreased GEnC activation and injury induced by neutrophils plus patient-derived PR3-ANCA-positive IgGs

**Fig. 5:**
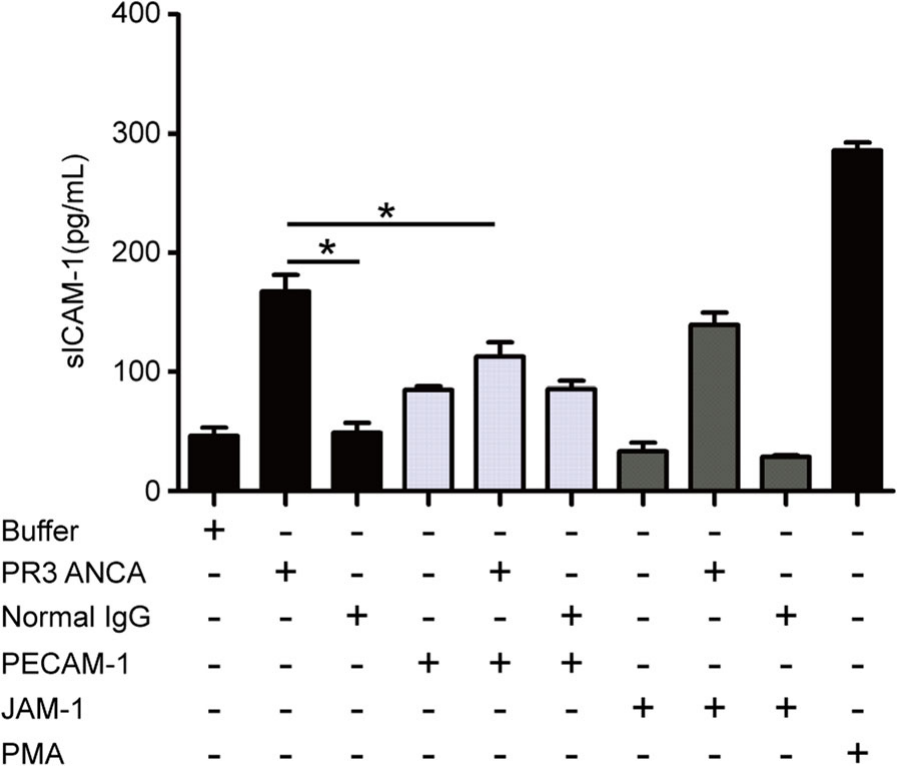
Platelet endothelial cell adhesion molecule-1 (PECAM-1) interaction with CD177 decreased GEnC activation and injury induced by patient-derived proteinase-3 (PR3)-antineutrophil cytoplasmic antibody (ANCA)-positive immunoglobulin (Ig)Gs (PR3 ANCA). By preincubation with PECAM-1, the levels of soluble intercellular cell adhesion molecule-1 (sICAM-1) significantly decreased in the supernatants of GEnCs treated with neutrophil plus patient-derived PR3-ANCA-positive IgGs. Bars denote means ± SD of sICAM-1 concentration (pg/mL). *p < 0.05. JAM-1 junctional adhesion molecule-1, PMA phorbol myristate acetate, TNF-α tumor necrosis factor-alpha

Soluble ICAM-1 is considered as one of the typical markers of endothelial cell activation and injury. Compared with GEnCs treated with TNF-α-primed neutrophils, the levels of sICAM-1 increased significantly in the supernatant of GEnCs treated with TNF-α-primed neutrophils plus patient-derived PR3-ANCA-positive IgGs (167.5 ± 27.7 pg/ml vs. 46.4 ± 14.5 pg/ml, *p* < 0.05). However, preincubation of neutrophils with PECAM-1 significantly decreased the level of sICAM-1 in the supernatant of GEnCs treated with primed neutrophils plus PR3-ANCA-positive IgGs (112.7 ± 24.2 pg/ml vs. 167.5 ± 27.7 pg/ml, *p* < 0.05) (Fig. 5).

## Discussion

Neutrophil transendothelial migration is a critical event in the inflammatory cascade, during which the heterophilic interaction between endothelial cell PECAM-1 and neutrophil CD177 plays an important role. PR3 can locate on neutrophils by binding to CD177, and it has been demonstrated that PR3-ANCA triggers degranulation and a respiratory burst of neutrophils by recognizing mPR3 and subsequently causes necrosis of endothelial cells [14]. The binding of CD177 with PR3 might be affected by the heterophilic interaction between endothelial cell PECAM-1 and neutrophil CD177. In this study, we demonstrated that sPECAM-1 could decrease mPR3 expression on neutrophils. We also found that PR3-ANCA-induced neutrophil activation and endothelial cell injury were alleviated by preincubation with PECAM-1. Therefore, role of CD177, the receptor of mPR3 on the neutrophil membrane, should be further depicted in the pathogenesis of AAV.

Besides its expression on endothelial cells as a major constituent of the endothelial cell intercellular junction, PECAM-1 could also be expressed on most cells of the hematopoietic lineage, including neutrophils [32]. During the process of neutrophil migration, endothelial PECAM-1 could interact with both PECAM-1 and CD177 on neutrophils. In the current study, sPECAM-1 was used to simulate endothelial PECAM-1 and to eliminate the influence from other adhesion molecules, e.g., selectins, on neutrophils and endothelial cells. Based on dissociation constants, the heterophilic interaction between CD177 and PECAM-1 is approximately 15 times stronger than the PECAM-1 homophilic interactions [11, 33], and the heterophilic interaction was further confirmed in our study. Considering the downregulating effect of sPECAM-1 on mPR3, we assumed that interaction of CD177 with its binding partner PECAM-1 may affect PR3 anchoring to the neutrophil membrane. However, the mechanism of the binding between CD177 and PR3 is not yet fully clear.

PR3-ANCA could bind and cross-link with mPR3 causing neutrophil activation [34, 35], which then contributes to necrotizing vasculitis. As reported, neutrophils with a higher level of mPR3 respond more strongly to PR3-ANCA in vitro, and the level of mPR3 expression correlates with disease severity [19, 36]. As detected in the current study, the level of mPR3 could be downregulated by the heterophilic interaction between CD177 and PECAM-1. Neutrophils with downregulated levels of mPR3 exhibited significantly lower levels of respiratory burst and degranulation in the presence of PR3-ANCA-positive IgGs. However, CD177 is a glycosylphosphatidylinositol (GPI)-anchored molecule that lacks an intracellular domain [10]; thus, intracellular signals could not be induced directly by CD177. As reported, CD177 could modulate neutrophil transmigration through activating CD11b/CD18 (Mac-1) [37], and neutrophil activation induced by PR3-ANCA could be attenuated by blocking CD177 or Mac-1 [38]. Therefore, PECAM-1 might attenuate PR3-ANCA-induced neutrophil activation by downregulating mPR3 as well as by inhibiting CD177 cross-linking with Mac-1.

In addition, SHP-1 phosphorylation was detected in neutrophils incubated with PECAM-1, indicating that both homophilic and heterophilic interaction might exist on neutrophils. However, the level of neutrophil activation induced by PR3-ANCA-positive IgGs was not obviously influenced by preincubation with sPECAM-1 in CD177-negative neutrophils, while it was significantly inhibited by sPECAM-1 in unsorted neutrophils. The results suggested that the inhibitory effect of PECAM-1 on neutrophil degranulation mainly resulted from the heterophilic interaction between CD177 and PECAM-1.

In AAV, endothelial cell injury is the result of the synergistic effect of several factors, including complement activation, neutrophil respiratory burst and degranulation, and neutrophil extracellular traps (NETs) release [14]. In our study, we demonstrated a protective effect of sPECAM-1 on endothelial cell injury by reducing neutrophil respiratory burst and degranulation in the presence of PR3-ANCA in vitro. However, PECAM-1 incubation could also elevate the level of free PR3 in the supernatant. Free PR3 could be acquired by endothelial cells, resulting in endothelial cytoskeleton disruption and subsequent apoptosis [39] which leads to a damaging effect on endothelial cells. Whether the protective effect outweighs the damaging effect of PR3 release in vivo is not clear.

## Conclusions

In conclusion, during the migration of neutrophils, PECAM-1 may interact with CD177 and decrease mPR3 expression on neutrophils, which results in attenuation of neutrophil activation and endothelial injury induced by PR3-ANCA. The current findings may have therapeutic implications in neutrophil-mediated PR3-ANCA vasculitis.

## Abbreviations

AAV: Antineutrophil cytoplasmic antibody-associated vasculitis
ANCA: Antineutrophil cytoplasmic antibody
BSA: Bovine serum albumin
DHR: Dihydrorhodamine
ECM: Endothelial cell basal medium
EDTA: Ethylenediaminetetraacetic acid
EGPA: Eosinophilic granulomatosis with polyangiitis
ELISA: Enzyme-linked immunosorbent assay
FITC: Fluorescein isothiocyanate
FSC: Forward scatter
GEnC: Glomerular endothelial cell
GPA: Granulomatosis with polyangiitis
HBSS: Hanks’ balanced salt solution
HRP: Horseradish peroxidase
Ig: Immunoglobulin
ITIM: Immunoreceptor tyrosine-based inhibitory motif
JAM-1: Junctional adhesion molecule-1
MACS: Magnetic cell sorting
MFI: Mean fluorescence intensity
MPA: Microscopic polyangiitis
MPO: Myeloperoxidase
mPR3: Membrane-bound proteinase-3
NaN_3_: Sodium azide
PBS: Phosphate-buffered saline
PE: Phycoerythrin
PECAM-1: Platelet endothelial cell adhesion molecule-1
PMA: Phorbol myristate acetate
PR3: Proteinase-3
ROS: Reactive oxygen species
sICAM-1: Soluble intercellular cell adhesion molecule-1
SSC: Side scatter
TNF-α: Tumor necrosis factor-alpha
